# Effects of Surface Properties of Fiber on Interface Properties of Carbon Fiber/Epoxy Resin and Its Graphene Oxide Modified Hybrid Composites

**DOI:** 10.3390/ma16114005

**Published:** 2023-05-26

**Authors:** Weihua Bai, Wenjun Liu, Weidong Li, Zewen Lin, Hong Qiu, Xiaolan Hu

**Affiliations:** 1 College of Materials, Xiamen University, Xiamen 361005, China; whuabai@163.com (W.B.); wenjun0807@126.com (W.L.); linzewen@stu.xmu.edu.cn (Z.L.); hqiu@xmu.edu.cn (H.Q.); 2 National Key Laboratory of Advanced Composites, AVIC Composite Technology Center, AVIC Composite Corporation Ltd., Beijing 101300, China; liwdhappy@163.com

**Keywords:** carbon fiber, surface property, interfacial property, composites, graphene oxide

## Abstract

In the present study, surface properties of three types of carbon fibers (CCF300, CCM40J, and CCF800H) on the interface properties of carbon fiber/epoxy resin (CF/EP) were analyzed. The composites are further modified by graphene oxide (GO) to obtain GO/CF/EP hybrid composites. Meanwhile, the effect of the surface properties of CFs and the additive graphene oxide on the interlaminar shear properties and dynamic thermomechanical properties of GO/CF/EP hybrid composites are also analyzed. The results show that the higher surface oxygen-carbon ratio of carbon fiber (CCF300) has a positive effect on improving the glass transition temperature (*T*_g_) of the CF/EP composites. The *T*_g_ of CCF300/EP is 184.4 °C, while the *T*_g_ of CCM40J/EP and CCF800/EP are only 177.1 °C and 177.4 °C, respectively. Furthermore, deeper and more dense grooves on the fiber surface (CCF800H and CCM40J) are more conducive to improving the interlaminar shear performance of the CF/EP composites. The interlaminar shear strength (ILSS) of CCF300/EP is 59.7 MPa, and that of CCM40J/EP and CCF800H/EP are 80.1 MPa and 83.5 MPa, respectively. For the GO/CF/EP hybrid composites, graphene oxide with abundant oxygen-containing groups is beneficial to improve the interfacial interaction. Graphene oxide can significantly improve the glass transition temperature and interlamellar shear strength of GO/CCF300/EP composites fabricated by CCF300 with a higher surface oxygen-carbon ratio. For the CCM40J and CCF800H with lower surface oxygen-carbon ratio, graphene oxide has a better modification effect on the glass transition temperature and interlamellar shear strength of GO/CCM40J/EP composites fabricated by CCM40J with deeper and finer surface grooves. Regardless of the type of carbon fiber, the GO/CF/EP hybrid composites with 0.1% graphene oxide have the optimized interlaminar shear strength, and the GO/CF/EP hybrid composites with 0.5% graphene oxide have the maximum glass transition temperature.

## 1. Introduction

Carbon fiber (CF) reinforced resin matrix composites play an important role in aerospace, rail transit, construction, and other fields because of their excellent properties, such as light-weight, high strength, and good structural designability [1,2,3]. As we all know, the interfacial bond formed by carbon fiber and resin is weak because of the high surface inertness of carbon fiber. Therefore, it is necessary to improve the interfacial interaction between carbon fiber and resin by means of surface activation [4,5], increasing the roughness and specific surface area of the fiber [6,7], etc. However, the results were not very satisfactory. Improving the interfacial interaction of carbon fiber/polymer composites is still an important research direction.

The research shows that the 0-dimensional particulate filler (alumina toughened zirconia, aluminum, and titanium, etc.) can strengthen the polymer with 1-dimensional carbon fiber to form a hybrid composite, which can effectively improve the mechanical properties of the material [8]. In recent years, 2-dimensional graphene-based nanomaterials have attracted extensive attention from researchers for their excellent physical and chemical properties. Graphene oxide (GO) is the oxide of graphene, and it contains a large number of hydroxyl, carboxyl, carbonyl, and epoxy groups containing reactive oxygen [9,10], resulting in good affinity and reactivity between graphene oxide and part of the thermosetting resin matrix [11,12,13]. In terms of epoxy resin (EP), the hydroxyl group and carboxyl group in graphene oxide can react with the epoxy group in epoxy resin to form a stable chemical bond, so the interface formed by graphene oxide and epoxy resin interacts well. Accordingly, the mixed emulsion formed by graphene oxide and epoxy resin was used for sizing carbon fiber, which could significantly improve the interfacial bonding strength of composite materials [14,15]. In addition, when GO is added to carbon fiber/epoxy resin composites, not only the bending strength and bending modulus of the composites can be effectively improved [16], but also the interlaminar shear properties, fracture toughness, and impact toughness of the composites can be significantly improved [16]. This means that graphene oxide has a significant impact on the interface properties of composites.

With the prosperity of the Chinese carbon fiber market, the performance of carbon fibers made in China are becoming more and more outstanding, gradually showing stronger and stronger competitiveness in the defense and civil fields. Currently, Chinese manufacturers have produced CCF300, CCM40J, and CCF800H carbon fiber products [17,18,19] corresponding to T300, M40J, and T800 of Toray. Accordingly, these carbon fibers reinforced resin matrix composites have also been developed gradually. For carbon fiber-reinforced resin matrix composites, carbon fiber surface treatment is indispensable. In general, surface sizing agent and groove treatment are different for different quality carbon fiber. Accordingly, the interfacial bonding of CF/polymer composites with different surface sizing agents and grooves is also different. The surface sizing of carbon fiber can change the surface polarity and surface energy of the fiber [20]. Moreover, the surface grooves of the fiber can also affect the interfacial shear properties of the fiber and the resin, and even have certain selectivity for the resin varieties [18]. In this paper, we analyzed the differences in the interface properties formed by epoxy resin and different types of carbon fibers. In order to further improve the interfacial properties of CF/EP composites, graphene oxide was used to modify the composites for fabricating the GO/CF/EP hybrid composites. To achieve this goal, we selected a typical high-temperature cured epoxy resin (E54 epoxy resin and diamino-diphenylsulfone (DDS)) as the matrix of the composite, and three kinds of Chinese carbon fibers, CCF300, CCM40J, and CCF800H as the reinforcing materials. The neat CF/EP and hybrid GO/CF/EP composites were prepared by wet prepreg technology and molding process. The properties of carbon fiber reinforced composites were investigated, and the relationship between the surface properties of carbon fiber and the interlayer shear properties and dynamic thermomechanical properties of the neat CF/EP and hybrid GO/CF/EP composites were mainly studied. This study deepens the understanding of the effect of surface oxygen-carbon activity and grooving of carbon fiber on the properties of carbon fiber/polymer and its GO modified hybrid composites, and contributes to the performance and structural design of high performance composites.

## 2. Experiments

### 2.1. Materials

**Figure 1:**
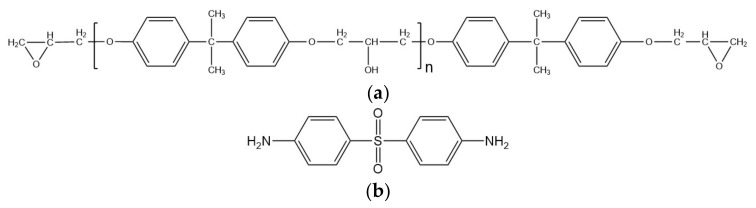
Chemical molecular structure formulas: (**a**) E54; (**b**) DDS.

H_2_SO_4_ (95~98%), HCl (36~38%) and NaNO_3_ were purchased from Xilong Scientific Co., Ltd. (Shantou, China). KMnO_4_ and H_2_O_2_ (30%) were purchased from Sinopharm Chemical Reagent Co., Ltd. (Shanghai, China). Graphite powder with 99% purity and 325 mesh, was purchased from Qingdao Huatai Lubrication Sealing Technology Co., Ltd. (Qingdao, China). The epoxy resin is bisphenol A glycidyl ether type epoxy resin (E54) with epoxy value 0.54 produced by Nantong Xingchen Synthetic Materials Co., Ltd., Wuxi, Jiangsu (Wuxi, China). Its chemical structure formula is shown in Figure 1a. The curing agent of epoxy E54 is 4,4’-diamino-diphenyl sulfone (DDS), a yellowish crystalline powder, produced by Shanghai Institute of Synthetic Resins (Shanghai, China). Its chemical structure formula is shown in Figure 1b. The carbon fibers, CCF300, CCM40J and CCF800H, used in this paper are all produced by Weihai Tuozhan Fiber Co., Ltd., (Weihai, China). CCF300 carbon fiber fabric is a satin fabric with a single layer density of (220 ± 7) g/m^2^. CCM40J carbon fiber fabric is a braided unidirectional curtain fabric with a single layer density of (190 ± 7) g/m^2^. CCF800H carbon fiber fabric is a braided unidirectional curtain fabric with a single layer density of (190 ± 7) g/m^2^.

### 2.2. Methods

Graphene oxide was prepared by the improved Hummers method [21], and a concentrated aqueous solution of graphene oxide was obtained. The graphene oxide aqueous solution was evenly stirred with ethanol, ultrasonic for 3 min, then centrifuged at 6000× *g* rpm for 30 min, and the supernatant was removed. The operation was repeated for 3 times. After that, acetone was added and centrifuged at 6000× *g* rpm for 30 min. The supernatant was poured out to obtain the lower layer, and the concentration of graphene oxide acetone dispersion was about 3.2 mg/mL.

E54-DDS epoxy resin glue was prepared according to the mass ratio of E54: DDS of 100: 33. The mass of graphene oxide was calculated according to 0.05%, 0.1%, 0.2%, 0.5%, 0.8%, 1.0%, 1.2%, 2% and 5% of the mass of E54-DDS. Graphene oxide acetone solution was added to E54-DDS acetone solution, stirred evenly, and ultrasound was carried out for 15 min to obtain GO-E54-DDS (GO-EP) acetone solution. The prepreg of carbon fiber fabric was prepared by wet method. The prepreg fabric was placed in the air for 72 h and then dried in a vacuum oven at 80 °C for 2 h to get the dry prepreg. The GO/CF/E54-DDS (GO/CF/EP) hybrid composites were prepared by moulding process, and the curing process condition was 140 °C/1 h + 160 °C/1 h + 180 °C/3 h. The GO/CF/EP composites with a thickness of about 2.0 mm and a carbon fiber volume fraction of 60–65% were obtained by heating curing and demoulding after cooling.

The neat CF/E54-DDS (CF/EP) composites were prepared according to the above process for comparison.

### 2.3. Characterization

The DSC 204 differential scanning calorimeter of NETZSCH, Selb, Germany was used for thermal analysis of the samples, and the heating rate was 10 °C/min. According to ASTM D 7028-2007e1 [22], the dynamic thermomechanical behavior of the sample was analyzed by DMA 242E dynamic thermomechanical analyzer of NETZSCH, Germany. The DMA sample size was 60 mm × 10 mm × 2 mm, the frequency was 1.0 Hz, and the heating rate was 5 °C/min. The DMA test temperature range was 30~300 °C, using double cantilever mode. According to GBT 30969-2014 [23], the interlaminar shear strength (ILSS) of the composites sample was tested. The ILSS sample size was 20 mm × 6 mm × 2 mm, and the test rate was 1.0 mm/min. SU-70 field emission scanning electron microscope of Japan Inc. (Tokyo, Japan). was used to observe the failure surface microstructure of the sample after the ILSS test, and the sample was treated with gold spray before the test. The cross-section crack morphology of the ILSS specimen was observed with Hitachi High-tech Co., LTD. (Tokyo, Japan) TM-3000 desktop scanning electron microscope. The surface morphology of carbon fiber was observed by Dimension Icon atomic force microscope (AFM) of Bruker, Billerica, MA, USA. The microstructure of graphene oxide was characterized by JEOL model JEM 2100 transmission electron microscope and SU-70 field emission scanning electron microscope of Japan Inc. (Tokyo, Japan). The graphene oxide powder was tested by the element analyzer Vario EL Ⅲ from German Elementar Company (Hanau, Germany), and the contents of elements N, C and H were obtained. Finally, the oxygen-carbon ratio of graphene oxide was calculated. Raman spectrum was measured by the HR Evolution confocal microscopic Raman spectrometer of Horiba, Paris, France, using 532 nm laser light source, spectral range was 3500 cm^−1^ to 600 cm^−1^, power was 5%, scanning time was 10 s. The characteristic absorption of graphene oxide and reduced graphene oxide was observed by D8-A25 X-ray diffractometer of Bruker, USA. The scanning range was from 5° to 90°, and the scanning step was 0.016°. The distribution of elements on the surface of carbon fiber was measured by PHI Quantum-2000 XPS photoelectron spectrometer (ULVAC-PHI, Chigasaki, Japan). The gel time was tested by manual wire drawing. The test tube was filled with about 2.5 g GO-EP pure glue. Controll the temperature and keep stirring. The time when the glue appeared wire drawing was recorded as the start time of gel, and the time when the wire drawing ended was recorded as the end time of gel.

## 3. Results

### 3.1. Carbon Fiber CCF300, CCF800H and CCM40J

**Figure 2:**
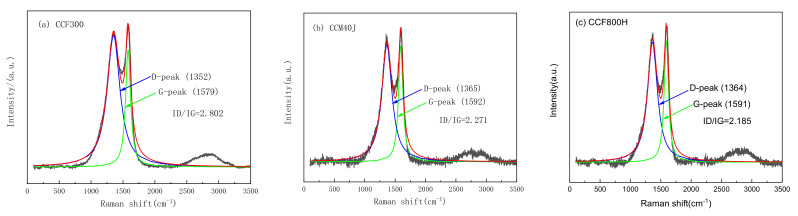
Raman spectra of carbon fibers: (**a**) CCF300; (**b**) CCM40J; (**c**) CCF800H.

The surface properties of carbon fiber mainly include surface physical properties and chemical properties. The physical properties of the surface mainly include surface morphology, grooves, and roughness. The surface chemical properties mainly include chemical composition, group type, and so on. The difference in surface properties of carbon fiber will affect the interface properties of composites. The basic properties of CCF300, CCM40J, and CCF800H are shown in Table 1. The results show that the density of CCF300, CCM40J, and CCF800H gradually increases, and the diameters of CCM40J and CCF800H are obviously smaller than that of CCF300. The tensile strength of CCF800H and the tensile modulus of CCM40J are the highest among the three carbon fibers. Figure 2 shows the Raman results of the three carbon fibers. Among them, 1352 cm^−1^ of CCF300, 1365 cm^−1^ of CCM40J, and 1364 cm^−1^ of CCF800H are their respective D-peaks. The 1579 cm^−1^ of CCF300, 1592 cm^−1^ of CCM40J, and 1591 cm^−1^ of CCF800H are their respective G-peaks. Raman curve results show that the ratio of D-peak to G-peak of CCM40J and CCF800H are 2.271 and 2.185, respectively, which are significantly smaller than the 2.802 of CCF300, indicating that the degree of graphitization and structure order of CCM40J and CCF800H are higher than that of CCF300. In addition, CCM40J and CCF800H have less disordered arrangement and less symmetrical carbon structure than CCF300, and the graphite lattice of CCM40J and CCF800H is more orderly [24]. Generally, CCM40J has a higher tensile modulus than CCF300 and CCF800H because it has a larger graphite microcrystal size [18] and a higher degree of graphitization [25].

**Figure 3:**
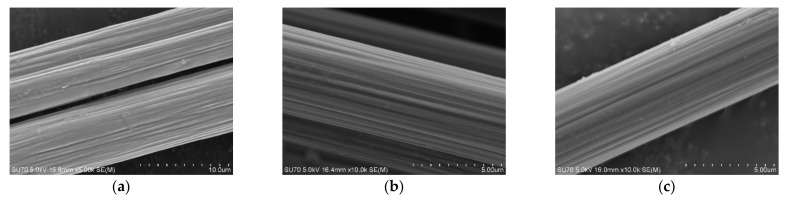
SEM pictures of surface morphology of carbon fibers: (**a**) CCF300; (**b**) CCM40J; (**c**) CCF800H.

**Figure 4:**
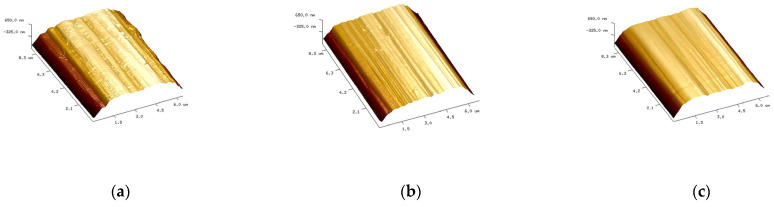
AFM images of surface morphology of carbon fibers: (**a**) CCF300; (**b**) CCM40J; (**c**) CCF800H.

The surface morphology of carbon fiber was characterized in Figure 3 and Figure 4 by SEM and AFM. The results show that the sizing agent can be observed on the surface of the carbon fibers. And the surface of the three fibers is etched to a certain extent, and the surface grooves are distributed along the axial direction of the fibers, with a certain roughness, as can be seen from Figure 3 and Figure 4. CCM40J and CCF800H have deeper and more dense grooves than CCF300. According to Figure 4 and the results in the literature [18,28], CCM40J indeed has deeper and more dense surface grooves than CCF300 and CCF800H.

**Figure 5:**
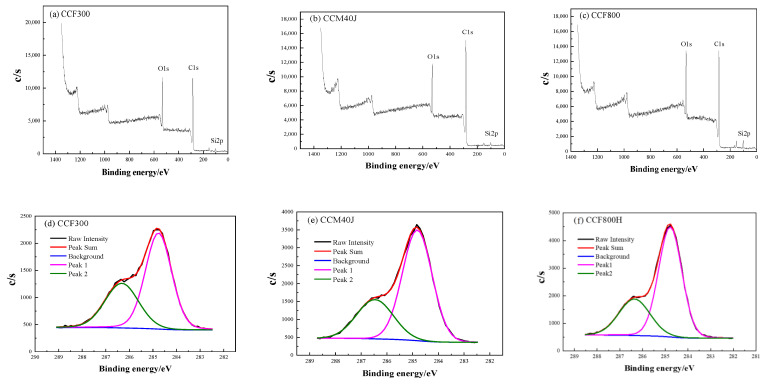
XPS broad spectrum of carbon fibers (**a**–**c**): (**a**) CCF300; (**b**) CCM40J; (**c**) CCF800H; and XPS C1s split-peak fitting curves (**d**–**f**): (**d**) CCF300; (**b**) CCM40J; (**f**) CCF800H.

Table 2 shows the results of surface element analysis for carbon fibers. After surface sizing, the surface oxygen-carbon ratio of CCF300 reaches 0.32. The surface oxygen-carbon ratios of CCM40J and CCF800H are 0.20 and 0.24, respectively. The element distribution test was further conducted on the surface of carbon fibers, and the results are shown in Figure 5. The X-ray photoelectron spectroscopy (XPS) spectra show that the fiber surface is mainly composed of carbon and oxygen element. The content of silicon and nitrogen is low, and the nitrogen peak cannot be clearly seen in the XPS spectrum. The results in Figure 5d–f show the partial peak fitting curves of XPS C1s of carbon fibers, and the partial peak fitting results are shown in Table 3. It can be seen from Table 3 that the surface oxygen-containing functional groups of the carbon fibers are all hydroxyl (−C−OH) or −C−OR, and no carbonyl group (−C=O) is detected. Generally, the thickness of the sizing agent layer reaches 50~100 nm, while the test depth of XPS is less than 10 nm, so the XPS result actually reflects the chemical information of carbon fiber surface sizing agents [29].

**Figure 6:**
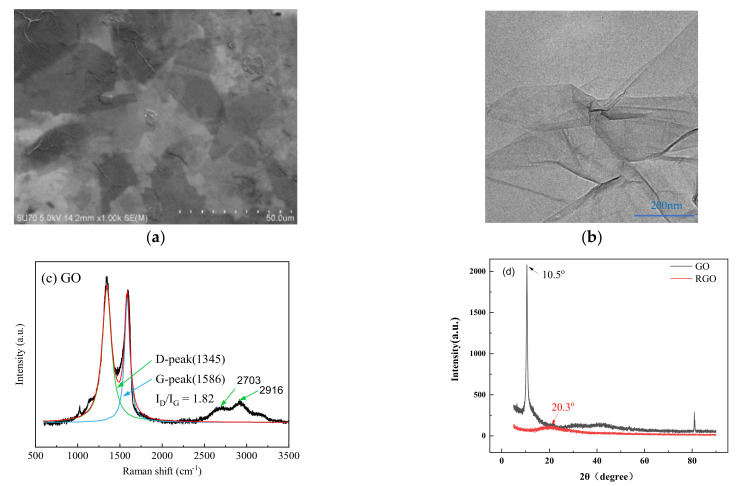
(**a**) SEM image of GO; (**b**) TEM image of GO; (**c**) Raman spectrum of GO; (**d**) XRD curve of GO and RGO.

Graphene oxide was prepared by hummers modified method [21]. The SEM photo of graphene oxide in Figure 6a shows that graphene oxide is laid flat on the substrate, showing a classic wrinkling appearance. This is attributed to the large surface area and high surface activity of the graphene oxide [30]. As can be seen from the TEM image of graphene oxide in Figure 6b, graphene oxide is thin and transparent with complete morphology. Figure 6c is the microscopic Raman spectrum of graphene oxide, in which 1345 cm^−1^ is the D-peak,1586 cm^−1^ is the G-peak. Unlike the small 2D peaks of graphene [31], GO has a strong D-peak, which also indicates that GO has more defects. These defects are mainly hydroxyl, carbonyl, carboxyl, and epoxide groups [9].

The graphene oxide goes through the composites preparation process, and the result in Figure 6d shows that the characteristic diffraction peak of the graphene oxide in the XRD curve changes from 10.5° to 20.3°, indicating that partial thermal reduction has taken place in graphene oxide, transforming it into reduced graphene oxide (RGO) [32]. Usually, when graphene oxide goes through the thermal molding process of composite materials, the high temperature will decompose the oxygen-containing functional groups of graphene oxide into gases such as CO, CO_2,_ and H_2_O. The rapidly escaping gases will generate huge pressure between the graphene oxide layers, and graphene oxide will expand and exfoliate under pressure, yielding reduced graphene oxide [33]. Thus, the GO in the composites will become the RGO.

**Figure 7:**
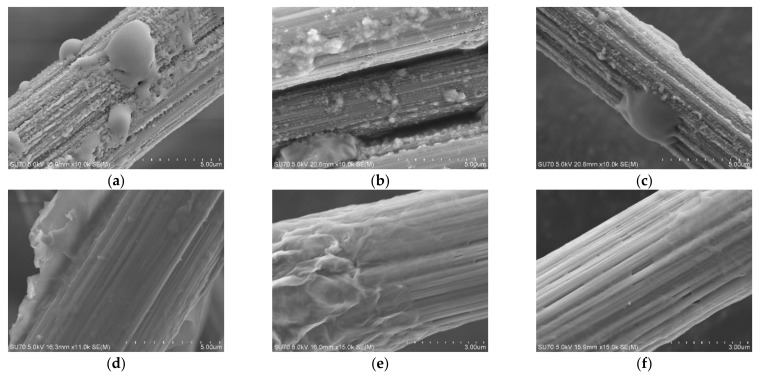
Surface morphology of carbon fiber infiltrated with pure epoxy resin (**a**–**c**): (**a**) CCF300-EP; (**b**) CCM40J-EP; (**c**) CCF800H-EP; and GO-EP composite glue (**d**–**f**): (**d**) CCF300-GO-EP; (**e**) CCM40J-GO-EP; (**f**) CCF800H-GO-EP.

Pure epoxy resin glue solution and GO-EP composite glue solution were used to impregnate carbon fiber bundles, and the morphology of the two types of glue on carbon fibers was compared and analyzed, as shown in Figure 7. As can be seen from Figure 7a–c, epoxy resin glue aggregates appeared on the surfaces of CCF300, CCM40J, and CCF800H, and granular epoxy resin glue could be observed on the fiber surfaces. However, as can be seen from the morphology of CCF300, CCM40J, and CCF800H impregnated with GO-EP composite glue in Figure 7d–f, GO-EP composite glue is evenly coated on the surface of the fibers. There is no resin aggregate on the surface of the fibers. The results in Figure 7 showed that the infiltration effect of GO-EP composite glue on carbon fibers was significantly better than that of pure epoxy resin glue. This indicates that the surface compatibility of GO-EP composite glue and carbon fiber has been significantly improved. This is attributed to graphene oxide’s large π-conjugated bond, thin lamellar structure, and abundant oxygen groups.

**Figure 8:**
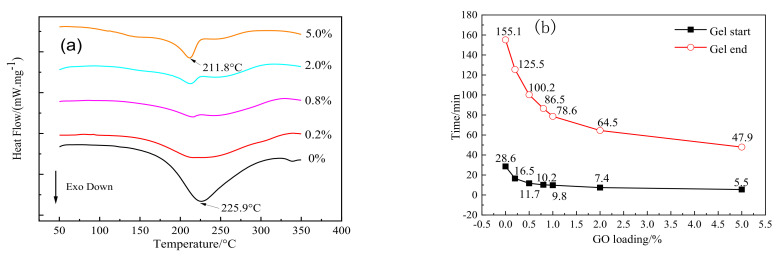
EP and GO-EP: (**a**) DSC curves; (**b**) gel curves at 120 ℃.

The heat curing performance of GO-EP composite resin was characterized by DSC and gel curves, and the results are shown in Figure 8 and Table 4. According to the DSC results in Figure 8, compared with pure epoxy resin, the initial reaction temperature and reaction exothermic peak temperature of GO-EP move to low temperature. The presence of hydroxyl and carboxyl groups can accelerate the addition curing reaction of epoxy and amine groups [34]. The hydroxyl and carboxyl groups in the graphene oxide structure can play a similar role, making the reaction of GO-EP move to a lower temperature than that of pure epoxy resin.

According to the gel results of GO-EP composite resin, at 120 °C, the gel start time of pure epoxy resin is 28.6 min, the gel end time is 155.1 min, and the gel opening window time is 126.5 min. With the increasing content of graphene oxide, the gel start time and end time of GO-EP were reduced gradually, and the gel opening window time decreased gradually. When the content of graphene oxide exceeded 2%, the changing trend of gel start time and gel end time became slower.

**Figure 9:**
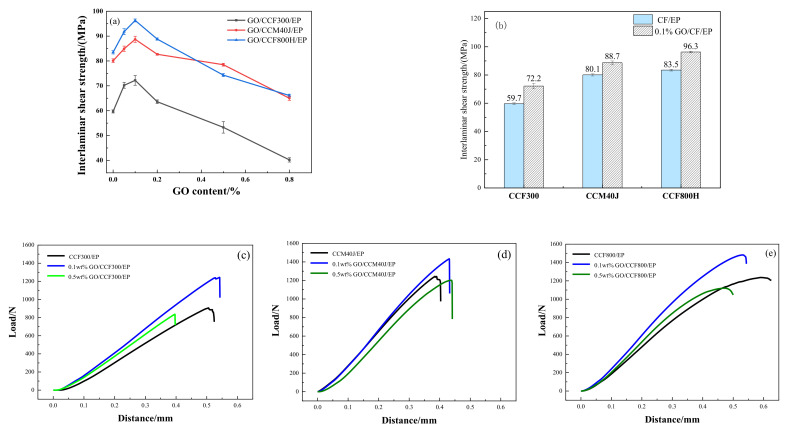
Results of interlaminar shear strength (ILSS) (**a**,**b**) and load distance curves (**c**–**e**) of the composites.

The pure epoxy glue and GO-EP composite glue were impregnated with carbon fiber, respectively, to prepare the corresponding neat CF/EP and hybrid GO/CF/EP composites. The interlaminar shear strength (ILSS) test was conducted on the composites, and the results are shown in Figure 9. For neat CF/EP composites (Figure 9a), the ILSS of CCF300/EP is the smallest (59.7 MPa), and that of CCM40J/EP and CCF800H/EP are 80.1 MPa and 83.5 MPa, respectively. Although the results in Table 2 and Table 3 show that the O/C ratio and the atomic concentrations of -C-OH and -C-OR are higher in CCF300, CCM40J/EP, and CCF800H/EP with lower O/C ratios, C-OH and -C-OR atomic concentrations (which are similar) show higher ILSS than CCF300/EP. This may benefit from the large number of grooves on the surface of the CCM40J and CCF800H fibers, as shown in Figure 4.

It seems that although surface grooves may bring defects and reduce the mechanical properties of fibers to a certain extent [35], grooves are beneficial to increase the specific surface area of the fibers, thus increasing the interface contact area and the mechanical meshing between the fiber and the matrix, resulting in improving the interface interaction [36,37]. This also indicates that the surface activity of the fiber does not play a decisive role in the ILSS of the composite materials, but the grooves on the fiber surface play a more important role in the ILSS.

With the addition of graphene oxide, the results in Figure 9a show that the ILSS of the GO/CF/EP hybrid composites increases with the increase of graphene oxide content when graphene oxide content is relatively low. When graphene oxide content is 0.1%, the ILSS of the corresponding GO/CF/EP hybrid composites reaches the maximum value, respectively. After that, ILSS of the GO/CF/EP hybrid composites decreased with the increase of graphene oxide content respectively. As shown in Figure 9b, the ILSS of 0.1%GO/CCF300/EP increased by 20.9% compared with that of the neat CCF300/EP, and that of 0.1%GO/CCM40J/EP increased by 10.7% compared with that of the neat CCM40J/EP. The ILSS of 0.1%GO/CCF800H/EP is 15.3% higher than that of the neat CCF800H/EP. In conclusion, CCF300, with a higher fiber surface activity, has a better performance than CCF800H and CCM40J in improving the interface properties of the corresponding GO/CF/EP hybrid composites.

Results in Figure 9c–e show that CCF800H/EP and CCM40J/EP have similar high shear failure loads. The failure distance of CCF800H/EP is significantly greater than that of CCM40J/EP, and the curve slope of CCM40J/EP is higher than CCF800H/EP and CCF300/EP. The failure distance of CCF300/EP is in the middle of the three neat CF/EP composites, but its shear failure load is the least. For the GO/CF/EP hybrid composites modified by 0.1%GO, the shear failure load of the corresponding 0.1%GO/CF/EP hybrid composites increases to varying degrees. 0.1%GO/CCM40J/EP has the smallest failure distance, while 0.1%GO/CCF800H/EP has the largest ILSS. Compared with the three GO/CF/EP hybrid composites, all corresponding GO/CCF300/EP showed the lowest ILSS as shown in Figure 9a.

**Figure 10:**
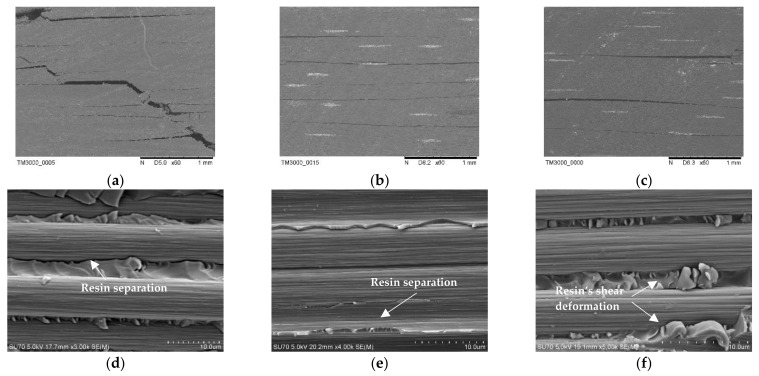
Microstructure of the neat CF/EP composites after interlaminar shear test: (**a**,**d**) CCF300/EP; (**b**,**e**) CCM40J/EP; (**c**,**f**) CCF800H/EP.

Figure 10 shows the macroscopic crack morphology of the neat CF/EP composites sample after the interlaminar shear performance test. Figure 10a shows that the main crack of CCF300/EP propagates laterally, with significant longitudinal penetration. Figure 10b-c shows that the cracks of CCM40J/EP and CCF800H/EP mainly propagate along the transverse direction, and the longitudinal propagation cracks are less than CCF300/EP. 

According to the failure surface microstructure of the neat CF/EP composites (Figure 10d–f), the carbon fiber surfaces are relatively smooth. There is an obvious separation between the epoxy resin and CCF300 fiber surface in CCF300/EP composites (Figure 10d). At the same time, the adhesion between resin and fiber surface in CCM40J/EP (Figure 10e) and CCF800H/EP (Figure 10f) is better than that in CCF300/EP. This is consistent with the results shown in Figure 9b that CCM40J/EP and CCF800H/EP have higher ILSS than CCF300/EP.

**Figure 11:**
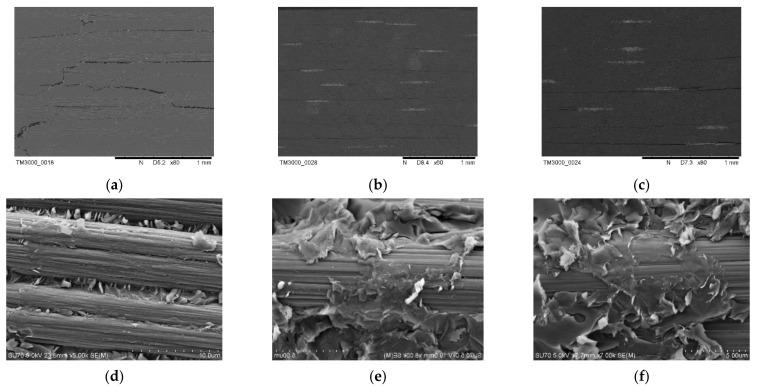
Microstructure of 0.1%GO/CF/EP hybrid composites after interlaminar shear test: (**a**,**d**) 0.1%GO/CCF300/E; (**b**,**e**) 0.1%GO/CCM40J/EP; (**c**,**f**) 0.1%GO/CCF800H/EP.

The microstructure of GO/CF/EP hybrid composites after the interlaminar shear performance test is shown in Figure 11. Figure 11a shows that there are obvious longitudinal cracks in the failure morphology of 0.1%GO/CCF300/EP. Figure 11b,c show that the failure morphology of 0.1%GO/CCM40J/EP and 0.1%GO/CCF800H/EP is mainly manifested as the transverse propagation crack, while the longitudinal crack is not obvious. 

Figure 11d shows that GO-EP composite resin adheres to the surface of CCF300 in 0.1%GO/CCF300/EP, and the adhesion and shear between resin and fiber are more obvious than that of the neat CCF300/EP. Figure 11e,f show that in 0.1%GO/CCM40J/EP and 0.1%GO/CCF800H/EP, the surface of carbon fiber is also obviously adhered to a lot of GO-EP composite resin. Meanwhile, it can be seen from Figure 11d,f that the internal crack growth direction of GO/CF/EP hybrid composite is very scattered and fine, indicating that the crack develops through the interface of graphene oxide and epoxy resin, forming a multi-directional dispersive crack. This phenomenon disperses the energy at the crack tip and is beneficial to improve the properties of the GO/CF/EP hybrid composites.

**Figure 12:**
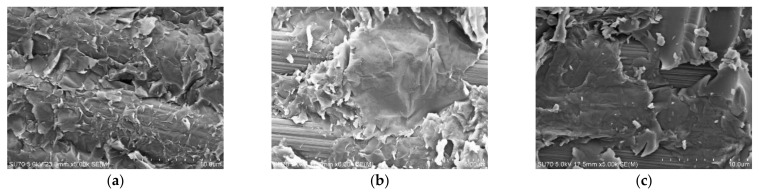
Microstructure of 0.5%GO/CF/EP hybrid composites after interlaminar shear test: (**a**) 0.5%GO/CCF300/EP; (**b**) 0.5%GO/CCM40J/EP; (**c**) 0.5%GO/CCF800H/EP.

With the further increase of graphene oxide content, the viscosity of GO-EP composite resin increased significantly, and it became difficult for the epoxy resin to impregnate graphene oxide and GO-EP composite resin to impregnate carbon fiber. In this case, graphene oxide is prone to aggregation. As shown in Figure 12, the failure morphology of 0.5%GO/CF/EP can be observed as the morphology of graphene oxide lamellar stripping. The van der Waals force between graphene oxide lamellae is weaker than the chemical bond force between graphene oxide and epoxy. When epoxy cannot infiltrate graphene oxide aggregates well, cracks are easy to start from graphene oxide lamellar, resulting in graphene oxide lamellar stripping failure, and then continue to cause and expand cracks, leading to material damage. As a result, the ILSS of GO/CF/EP hybrid composites with a large amount of graphene oxide is decreased, as shown in Figure 9a.

### 3.2. Dynamic Thermomechanical Properties

**Figure 13:**
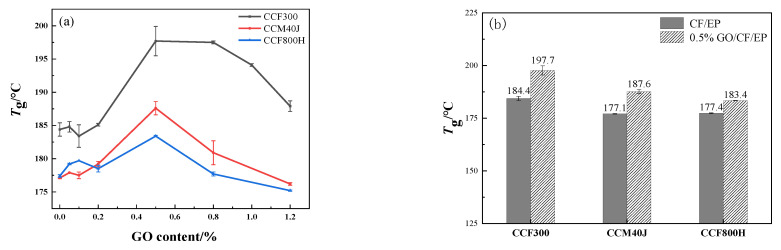
*T*_g_ results of dynamic thermomechanical properties of composites, (**a**) *T*_g_ results of the GO/CF/EP composites, (**b**) *T*_g_ results of 0.5%GO/CF/EP.

The dynamic thermomechanical properties of the composites were characterized and shown in Figure 13. Figure 13a,b show that the resin matrix is also E54-DDS, and the glass transition temperature (*T*_g_) of CCF300/EP is 184.4 °C, while the *T*_g_ of CCM40J/EP and CCF800H/EP is only 177.1 °C and 177.4 °C, respectively. The *T*_g_ value measured in the composite materials is actually obtained by the superposition of the resin matrix and the interface interaction between the resin and the fiber [38]. The result in Figure 13a shows that the interface interaction of CCF300/EP is stronger than that of CCM40J/EP and CCF800H/EP. This indicates that the high oxygen-carbon ratio on CCF300 surface has a greater enhancement on the interfacial interaction between fiber and resin in CCF300/EP composites.

Figure 13a also shows that the *T*_g_ values of GO/CF/EP hybrid composites have little change compared with the corresponding neat CF/EP when graphene oxide content is less than 0.2%. However, the *T*_g_ values of 0.5%GO/CF/EP are significantly larger than those of corresponding neat CF/EP. According to the *T*_g_ increase of the composites (Figure 13b), CCF300 with a higher surface oxygen-carbon ratio has an obvious advantage over CCM40J and CCF800H. This indicates that the abundant oxygen-containing groups in graphene oxide strengthen the chemical bonding and physical action with the active groups on the surface of CCF300, which is conducive to improving the *T*_g_ of the composite materials. The *T*_g_ levels of the three corresponding GO/CF/EP hybrid composites are all significantly increased, which also indicates that the abundant oxygen-containing groups of graphene oxide can effectively enhance the interface interaction of GO/CF/EP hybrid composites.

In view of the optimum interlaminar shear strength of 0.1%GO/CF/EP and the optimum glass transition temperature of 0.5%GO/CF/EP, a small amount (0.1%) of graphene oxide is beneficial to the infiltration of epoxy into graphene oxide and the good dispersion of graphene oxide in the resin, which is conducive to the improvement of the interlaminar shear performance of the composites. However, a relatively large amount of graphene oxide (0.5%) is more conducive to the formation of more chemical bonds and the improvement of the cross-linking density of the GO-EP composite resin due to the more oxygen-containing groups of graphene oxide in the GO-EP composite resin, which is more beneficial to the improvement of the heat resistance of the GO/CF/EP hybrid composites.

**Figure 14:**
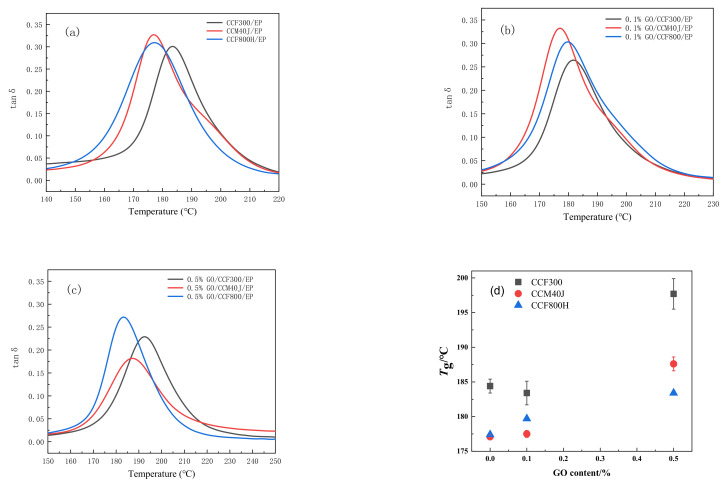
Tanδ values of dynamic thermomechanical properties of the composites: (**a**) neat CF/EP; (**b**) 0.1%GO/CF/EP; (**c**) 0.5%GO/CF/EP; (**d**) *T*_g_.

The dynamic thermomechanical performance curve and the mechanical loss factor (tanδ) reflect the material damping characteristics [38]. For carbon fiber-reinforced composites, the motion of interfacial molecular chains can reflect the damping of the materials. The tanδ value of the composites is smaller, showing that the bondage of fiber on molecular chain segments is more obvious, indicating stronger interface bondability [39,40]. The results of neat CF/EP in Figure 14a show that the tanẟ value of CCF300/EP is the least in all CF/EP composites. And the tanδ value of CCM40J/EP is slightly higher than that of CCF800H/EP. Combined with the surface oxygen-carbon ratio results of the carbon fibers in Table 2, one can see that the high surface oxygen-carbon ratio of carbon fiber is conducive to the strengthening of the interface interaction between the fiber and the resin matrix. This also corroborates the results in Figure 13.

The results in Figure 14b,d indicate that 0.1%GO/CCF300/EP has the largest *T*_g_ and smallest tanδ value within all corresponding 0.1%GO/CF/EP composites. Compared with the CCM40J/EP, the *T*_g_ value of 0.1%GO/CCM40J/EP is almost no increase, while the tanδ value of 0.1%GO/CCM40J/EP is the highest of the three corresponding GO/CF/EP hybrid composites. And the *T*_g_ appreciation and tanδ value of 0.1%GO/CCF800H/EP are medium.

Results in Figure 14c show that the *T*_g_ value of 0.5%GO/CCF800H/EP only increased by 6 °C than that of the neat CCF800H/EP, and it also shows the highest tanδ value of the three corresponding neat CF/EP composites. The results in Figure 14c also indicate that, compared with the neat CF/EP and 0.1%GO/CF/EP, the tanδ values of 0.5%GO/CF/EP composites are reduced. It also shows that the interface interaction of the three 0.5%GO/CF/EP hybrid composites is enhanced correspondingly.

## 4. Conclusions

Three kinds of Chinese carbon fibers, CCF300, CCM40J, and CCF800H, with different surface properties, were used as the reinforcement materials of the carbon fiber/epoxy resin (CF/EP) composites. The effects of the surface oxygen-carbon ratio and grooves of carbon fiber on the interfacial properties of the composites were mainly characterized by interlaminar shear strength and dynamic thermomechanical properties. The results show that the surface oxygen-carbon ratio of carbon fiber significantly affects the glass transition temperature (*T*_g_) of the neat CF/EP composites. The composites fabricated by CCF300 with a higher surface oxygen-carbon ratio have a higher *T*_g_ than CCM40J and CCF800H with a smaller surface oxygen-carbon ratio. The *T*_g_ of CCF300/EP is 184.4 °C, while the *T*_g_ of CCM40J/EP and CCF800/EP are only 177.1 °C and 177.4 °C, respectively. Meanwhile, the deep and fine grooves on the surface of carbon fiber are more favorable to the improvement of the interlaminar shear performance of the neat CF/EP composites. The composites fabricated by CCM40J and CCF800H with deeper and finer grooves show a higher interlaminar shear strength than CCF300. The interlaminar shear strength (ILSS) of CCF300/EP is 59.7 MPa, and that of CCM40J/EP and CCF800H/EP are 80.1 MPa and 83.5 MPa, respectively.

The CF/EP composites were modified by graphene oxide (GO). The results show that the fiber surface wettability of GO-EP composite resin for carbon fiber is significantly improved than that of pure epoxy resin. The application of graphene oxide enhances the interface interaction of GO/CF/EP hybrid composites. The lower content of graphene oxide (e.g., 0.1%) is more conducive to the improvement of the interlamellar shear performance of GO/CF/EP hybrid composites, while the higher content of graphene oxide (e.g., 0.5%) is more beneficial to the improvement of the *T*_g_ of GO/CF/EP hybrid composites. The ILSS of 0.1%GO/CCF300/EP, 0.1%GO/CCM40J/EP, and 0.1%GO/CCF800H/EP increased by 20.9%, 10.7%, and 15.3% compared with their corresponding CF/EP, respectively. Furthermore, the *T*_g_ of 0.5%GO/CCF300/EP, 0.5%GO/CCM40J/EP, and 0.5%GO/CCF800H/EP are respectively 13.4 °C, 10.5 °C, and 6.0 °C higher than those of the corresponding CF/EP. Graphene oxide has a better modification effect on the composites reinforced by CCF300 with a higher surface oxygen-carbon ratio, both for interlaminar shear performance and glass transition temperature. While CCM40J and CCF800H with lower surface oxygen-carbon ratios, graphene oxide has a better modification effect on the composites reinforced by CCM40J with deeper and finer grooves.

## Figures and Tables

**Table 1:** Basic properties of carbon fibers.

Carbon Fiber	Tensile Strength /GPa	Tensile Modulus /GPa	Density /(g·cm^−3^)	Diameter /μm
CCF300 [26]	3.90	220	1.78	7.0
CCM40J [25]	4.41	377	1.79	5.0
CCF800H [27]	5.49	290	1.81	5.3

**Table 2:** Surface element analysis of carbon fibers.

Carbon Fiber	C1s	O1s	Si2p	N1s	O/C Ratio
	Content/%	Content/%	Content/%	Content/%	
CCF300	72.93	23.24	3.09	0.74	0.32
CCM40J	80.93	15.99	2.27	0.81	0.20
CCF800H	76.03	18.16	4.95	0.86	0.24

**Table 3:** Peak fitting results of carbon fibers.

Carbon Fiber	Peak 1 (−C−C or −C−H)		Peak 2 (C−O)	
	BE/eV	Content/%	BE/eV	Content/%
CCF300	284.8	63.2	286.3	36.8
CCM40J	284.8	69.7	286.5	30.3
CCF800H	284.8	69.3	286.4	30.7

**Table 4:** DSC data of EP and GO-EP.

Sample	Initial Reaction Temperature (*T*_i_/°C)	Exothermic Peak Temperature (*T*_p_/°C)	Termination Reaction Temperature (*T*_d_/°C)	Enthalpy of Reaction (∆H/J.g^−1^)
Pure EP	181.5	225.9	274.2	−359.5
0.2% GO-EP	172.1	221.9	293.2	−339.0
0.8% GO-EP	165.1	215.6	282.8	−191.6
2.0% GO-EP	121.0	212.7	294.5	−162.3
5.0% GO-EP	112.8	211.8	285.2	−190.4

## Data Availability

Not applicable.
